# Presence of *Helicobacter pylori *in betel chewers and non betel chewers with and without oral cancers

**DOI:** 10.1186/1472-6831-9-23

**Published:** 2009-09-22

**Authors:** Neluka Fernando, Gnanapragasam Jayakumar, Naomal Perera, Indranee Amarasingha, Fahra Meedin, John Holton

**Affiliations:** 1 Department of Microbiology, Faculty of Medical Sciences, University of Sri Jayewardenepura, Sri Lanka; 2 General Hospital Badulla, Sri Lanka; 3 Cancer Institute Maharagama, Sri Lanka; 4 Centre for Infectious Disease and International Health, Windeyer Institute of Medical Sciences, Royal Free & University College London Medical School, UK

## Abstract

**Background:**

Betel chewing has been shown to predispose to periodontal disease and oral cancer. Studies show that people with gum disease are more likely to test positive for *Helicobacter pylori (H. pylori)*. It is not known if the lesions produced by betel quid and the resulting, chemical changes predispose to colonization by *H. pylori*. Further the role of this organism in oral cancer is not known. Our objective was to determine the presence of *H. pylori *in oral lesions of thirty oral cancer patients and to determine the presence of IgG antibodies to *H. pylori *in oral cancer patients who are betel chewers and non betel chewers, healthy betel chewers and healthy non-betel chewers and to compare the presence of *H*. *pylori *in these four groups. This case control study was conducted at the Cancer Institute Maharagama and the Department of Microbiology, Faculty of Medical Sciences, University of Sri Jayewardenepura.

**Methods:**

One hundred and seventy three subjects, of whom fifty three were patients presenting with oral cancer to the Cancer Institute Maharagama, sixty healthy betel chewers and sixty healthy non-betel chewers from the Religious and Welfare Service Centre Maharagama were tested for *H. pylori *by serology. Thirty oral biopsies from oral cancer patients were cultured under microaerophilic condition to isolate *H. pylori*. The statistic used was Chi-square test.

**Results:**

Of the fifty-three oral cancer patients, forty-four were betel chewers. Among the 53 oral cancer patients examined, ten of forty-four (10/44 = 22.7%) patients who are betel chewers and four of nine (4/9 = 44.4%) patients who are non-betel chewers were detected positive for IgG antibody against *H. pylori*. In the healthy group (betel chewers and non betel chewers) ten (16.7%) of the healthy betel chewers tested positive for *H. pylori *by serology. None of the healthy non-betel chewers tested positive for *H. pylori*

Fourteen [26.4%] of oral cancer patients tested positive for *H. pylori *by serology, of which two were also culture positive (Only thirty samples were cultured). The presence of *H. pylori *in betel chewers (with or without cancer) compared to non-betel chewers was statistically significant. (Chi-square test p < 0.05) The use of tobacco and areca nut in betel chewers was significant with the presence of *H. pylori *(p < 0.05).

**Conclusion:**

There is a significant higher proportion of *H. pylori *in betel chewers compared to non-betel chewers but not between oral cancer patients compared to patients without oral cancer. Hence Betel chewing may predispose to colonisation with *H. pylori *in the digestive tract through swallowing the quid or during betel chewing.

## Background

*Helicobacter pylori *is a micro-aerophilic bacterium found principally in the stomach [1]. Infection with this organism is widespread, including Sri Lanka [2-4] and epidemiological studies have clearly demonstrated a major etiological role of *H. pylori *for peptic ulcer disease, gastric MALT [mucosal associated lymphoid tissues] lymphoma, and distal gastric cancer [5,6]. A number of virulence characteristics have been identified that may be linked to the development of ulcers and cancer in the stomach. The organism possesses a urease enzyme that increases the local concentration of ammonium ion (a cytotoxin); it produces a vacuolating cytotoxin (vacA), which leads to apoptosis and necrosis; it injects a protein (cagA) into host cells, which affects intracellular signalling events. The net results of these and other virulence characteristics are the development of ulcers and altered cell cycle events that are implicated in the development of gastric cancer [7].

There are conflicting results reported in the literature on the isolation of *H. pylori *from dental plaque. Several studies indicate a low prevalence of *H. pylori *in the oral cavity of their patients and consider that it is not a significant environment for this bacterium [8,9]. Some studies suggest that *H. pylori *has only a transient presence in the oral cavity and also demonstrate the antagonist effects of some oral bacteria to *H. pylori*, which could inhibit colonization by this organism in the oral cavity [10,11]. On the other hand, authors who found this bacterium in almost all of their study population consider that the oral cavity may act as a reservoir for re-infection of the stomach and that *H. pylori *is part of the normal micro biota in the mouth [12-14].

Oral cancer represents approximately 3% of all cancers in the world and ranks 6^th ^globally. Fifty eight percent of oral cancers are concentrated in South and South-East Asia with Pakistan having the highest reported incidence [15]. Oral cancer is also common in the Sri Lankan population. According to the cancer register in Sri Lanka the Island wide incidence of oral specified and unspecified, oro-pharynx cancer for the year 2000 is 14.6%.

Epidemiological studies have revealed that betel quid chewing is a popular habit in Asian countries; it is a very common habit in Sri Lanka, which is associated with an increased risk of oral cancer and oral sub mucous fibrosis [16]. The composition of betel quid varies with different geographical locations however the general constituent of quid is areca nut [*Areca catechu*] betel leaf, lime with or without tobacco. Studies have also shown a high prevalence of periodontal disease among betel quid chewers as compared to non-betel quid chewers [17,18,30]. Poor plaque control might explain the higher prevalence of periodontal disease in betel quid chewers [19]. Other risk factors for oral cancer are smoking, and alcohol consumption [18,19,31]

As *H. pylori *is classified as a Class I carcinogen it is relevant to know whether the lesions produced by betel chewing and the resulting chemical changes that take place in oral cavity may facilitate the colonization by *H. pylori *thus leading to carcinogenesis. The role of this organism in predisposing to oral cancer is not known. Therefore our aim was to study the presence of *H.pylori *in oral cavity of a Sri Lankan population with oral cancer and whether betel chewing may facilitate colonization of this organism thus enhancing the carcinogenic effect of tobacco and areca nut, which are the main constituents of betel quid.

In this article we show that betel chewing predisposes to colonisation by *H. pylori *but that there does not appear to be an association with oral cancer.

## Methods

One hundred and seventy three subjects [119 males; 54 females] within the age group 30-70 years were enrolled in the study. Fifty three subjects were patients with oral cancer from the Cancer Institute Maharagama Sri Lanka, sixty healthy betel chewers and sixty healthy non-betel chewers were from the Religious And Welfare Service Center, Maharagama, Sri Lanka. The study was approved by the Ethics Committee of Faculty of Medical Sciences, University of Sri Jayewardenapura. Exclusion criteria were patients who had endoscopic evidence of gastritis or gave a history of gastric cancer or peptic ulcer disease, treatment with H2-receptor antagonist, proton pump inhibitors or antibiotics within the preceding six months. Subjects were excluded from the healthy non-betel chewer subgroup if they had consumed even one pack of betel at any time.

All subjects were given a questionnaire, recording demographic data, cigarette smoking, alcohol consumption, oral hygienic practices and sleeping with quid in the mouth during night.

Oral biopsies from thirty oral cancer patients were cultured under micro aerophilic conditions to isolate *H. pylori*. A biopsy from each patient was placed in a special transport medium for *H. pylori *[Portagerm, France] respectively and brought to the laboratory with minimum delay. The biopsies were inoculated onto blood agar containing Selective Campylobacter Supplement (Skirrow) [Oxoid, UK] and incubated at 37°C in a micro-aerobic environment for four days. *H. pylori *was identified by routine biochemical methods.

Five mls of blood was collected from each of the subject in the four groups; The blood was centrifuged at 3500 rpm for 15 minutes and supernatant serum sample separated and stored at -80°C. *H. pylori *IgG antibodies in serum were detected by a commercial enzyme linked immunosorbant assay [Novum Diagnostica, UK] according to the manufacture's instructions. Absorbance values were recorded using a micro- titre plate reader with a 450 nm filter, and results were calculated according to the manufacturer's instructions. This test has sensitivity of >95% and specificity of >95%. The betel chewers and non-betel chewers were not biopsied due to ethical issues.

### Statistical Analysis

Statistical analysis was performed using the Chi-square test

## Results

The association between presence of *H. pylori *in oral cancer patients who are betel chewers, non betel chewers and healthy betel chewers and non betel chewers are shown in Table 1. Of the fifty-three oral cancer patients, forty four were betel chewers. Among the 53 oral cancer patients examined, ten of forty-four (10/44 = 22.7%) patients who are betel chewers and four of nine (4/9 = 44.4%) patients who are non-betel chewers were detected positive for IgG antibody against *H. pylori*. In the healthy group (betel chewers and non betel chewers) ten (16.7%) of the healthy betel chewers tested positive for *H. pylori *by serology. None of the healthy non-betel chewers tested positive for *H. pylori *(Table 1).

**Table 1:** Detection of serum IgG against *H. pylori *in patients with oral cancer(betel chewers/non betel chewers) compared to healthy control subjects (betel chewers/non betel chewers)

***H. pylori***	**Group 1** **Oral cancer patients betel chewers**	**Group 2** **Oral cancer patients non betel chewers**	**Group 3** **Healthy Betel chewers**	**Group 4** **Healthy Non-betel chewers**	**Total of Groups 1-4**	**Detection of *H.pylori *IgG in betel chewers** **(Gps.1 & 3)**
Positive	10 (22.7%)	4 (44.4%)	10 (16.7%)	0	24	20

Negative	34 (77.3%)	5 (55.6%)	50 (83.3%)	60 (100%)	149	84

Total	44	9	60	60	173	104

Of fourteen (26.4%) oral cancer patients (betel chewers and non betel chewers) who tested positive for *H. pylori *by serology, two were culture positive (only thirty samples were cultured) and they were also betel chewers.

Of the 104 betel chewers (with or without cancer) 20 (19.2%) were *H. pylori *positive and half of them were oral cancer patients (10/20) whereas the other half were healthy subjects (10/20). When compared to betel chewing healthy controls, the presence of serum IgG against *H. pylori *in Betel chewing oral cancer patients was not statistically significant. (chi square test p > 0.05) - Table 1.

There was a significant difference in presence of *H. pylori *in betel chewers (with or without cancer) compared to non-betel chewers (with or without cancer) (20/104 and 4/69 respectively; Chi-square test) (p < 0.05) - Table 2.

**Table 2:** Presence of Serum IgG against *H. pylori *in Betel chewers and Non Betel chewers

	**Betel Chewers**	**Non Betel Chewers**	**Total**
H.pylori positive	20 (19.2%)	4 (5.8%)	24

H.pylori negative	84 (80.8%)	65 (94.2%)	149

Total	104	69	173

X^2 ^= 15.0, (p < 0.05); Significant

The association between presence of serum IgG against *H. pylori*, in betel chewers (with or without cancer) in relation to other factors are shown in Table 3. The use of tobacco and areca nut in betel chewers was significant with the presence serum IgG against *H. pylori *(p < 0.05). There was no significant difference in the presence of serum IgG against *H. pylori *in betel chewers and their practices such as swallowing the quid, sleeping with quid during the night and washing the mouth after chewing betel. Further there was no significant difference in consumption of alcohol or smoking with the presence of serum IgG against *H pylori *in all four groups (p > 0.05).

**Table 3:** Presence of serum IgG against *H pylori *in Betel chewers (oral cancer patients and healthy betel chewers) in relation to other factors

	***H pylori *positive (n = 20)**	***H pylori *negative (n = 84)**	**Chi-square test** **(Table value 3.84;p = 0.05)**
Use of tobacco and areca nut	19 [95%]	53 [63.1%]	p < 0.05 significant

Swallowing the quid	4 [20%]	14 [16.7%]	p > 0.05 Not significant

Sleeping with quid during night	10 [50%]	4 [4.8%]	p > 0.05 Not significant

Washing the mouth routinely after chewing betel	12 [60%]	62 [73.8%]	p > 0.05 Not significant

Of the 119 males seventeen (14.3%) were tested positive for serum IgG against H. pylori and of the 54 females, seven (13%) were detected positive for serum IgG against H. pylori. Hence there is no significant difference in the presence of antibodies against H. pylori between males and females. Table 4.

**Table 4:** Detection of *H. Pylori *antibodies between betel chewing males and females and between the age groups 31-50 yrs and 51-70 yrs: Males - 119; Females - 54;

	**Oral cancer betel chewers**	**Oral cancer non betel chewers**	**Healthy betel chewers**	
**Males**				
*H.pylori *+ve	7	3	7	17 (14.3%)
*H.pylori -ve*	31	3	39	73 (61.1%)

**Females**				
*H.pylori +ve*	3	1	3	7 (13.0%)
*H.pylori -ve*	3	2	11	16 (29.6%)

**31-50 yrs**				
*H.pylori +ve*	1	1	6	8(9.4%)
*H.pylori -ve*	10	1	34	45 (53.0%)

**51-70 yrs**				
*H.pylori +ve*	9	3	4	16 (18.2%)
*H.pylori -ve*	24	4	16	44 (50.0%)

## Discussion

In the present study, the association between oral cancer patients chewing betel and non betel chewers, healthy betel chewers and non betel chewers and the presence of serum IgG against *H. pylori *were evaluated.

The results demonstrate that the proportion of serum IgG against *H. pylori *was higher in betel chewers (Oral cancer patients and healthy subjects) compared to healthy non-betel chewers, and this was statistically significant.

A study of 20 patients from the UK with oral cancer, using both culture and 16S rDNA amplification of superficial and deep tumour tissue demonstrated a wide range of organisms, none of which were *Helicobacter *species [20]. As betel chewing is not at all common in the UK it is reasonable to suppose that all these patients did not chew betel. The effect on the oral flora of oral cancer and chewing tobacco has previously been studied. A study in patients with oral carcinoma showed that the number of micro-organisms on the surface of oral lesions was higher than that of healthy mucosa [21] and a study of the effects of chewing tobacco (gutkha) in a population in India showed that both chewing and smoking tobacco reduced salivation and altered the normal oral microflora [22]. Some previous studies have investigated the presence of *H. pylori *in oral cancer. A study of *H. pylori *in gastric and oral specimens from patients with peptic ulcer disease and oral cancer showed that *H. pylori *could be detected in 12% of oral specimens and that growth of *H. pylori *was inhibited by oral microflora [10]. Similarly, a study of patients with benign oral ulcers and oral cancer showed that *H. pylori *could be detected in 11-15% depending on the method used although the organism could not be cultured and the authors suggested that there was no association between the oral lesions and *Helicobacter pylori *[23].

As far as we are aware the interaction between betel chewing, oral cancer and colonization by *H. pylori *as hitherto not been investigated.

Previously published data show a number of oral diseases associated with betel chewing [24,25] principally oral sub mucous fibrosis and oral carcinoma. There is evidence that areca nut extracts inhibit growth, attachment and matrix protein synthesis of cultured gingival fibroblasts, which supports the concept that betel quid chewing may affect periodontal health and thus predispose to colonization [26]. In addition areca nut extract has been shown to interfere with the action of neutrophils, again suggesting that areca nut promote the bacterial colonization and periodontal disease [27]. In vitro evidence suggests that areca nut extracts may suppress the growth of several oral micro organisms [28]. Modulation of the normal periodontal defences could provide a favourable environment for colonization of *H. pylori*. This is supported by previous study by Gebara et al [29], who showed a high prevalence of *H. pylori *infection in patients with periodontitis.

The fact that *H. pylori *was more commonly seen in betel chewers, compared to non-betel chewers and statistically significant, in this study, supports this hypothesis. Further in this study it was noted that out of 44 oral cancer betel chewing patients 10 tested positive for serum IgG against *H. pylori*. Among the betel chewers those using both tobacco and areca nut were significantly associated with presence of *H. pylori *supporting the hypothesis that both tobacco and areca nut can modulate the periodontal defences thus favouring the colonization of this organism.

According to epidemiological studies the site of the infection of *H. pylori *may vary. Possible places for the colonization of *H. pylori *may be stomach or oral cavity or both. Antibodies will be produced as a result of a systemic immune response against this pathogen. In this study we have detected the presence of *H. pylori *antibodies in the serum of patients and healthy betel chewers but not in healthy non-betel chewers. We confirmed that the antibodies for this organism that were detected in the serum were only due to infection of *H. pylori *in the oral cavity with the exclusion of *H. pylori *infection of stomach. This confirmation was done by upper gastrointestinal endoscopies in all the oral cancer patients but not in the healthy controls. To further confirm the results, biopsies were tested by culture from thirty of the oral lesions of oral cancer patients. Due to ethical reasons we did not take biopsies from the control groups. In all groups however a history of treatment with H2-receptor antagonists, proton pump inhibitors or antibiotics for the preceding six months was an exclusion criteria Although a statistical significance between betel chewers and non betel chewers were shown only by serology, it is possible to conclude that the site of colonization of *H pylori *is indeed the oral cavity due to changes induced in oral mucosa by betel chewing as it was significantly more in betel chewers when compared to non betel chewers.

## Conclusion

An important observation of our study is that 19.2% of all betel chewers were *H. pylori *positive as compared with non-betel chewers (5.8%). The implication of this finding is that betel quid has some effects on providing a favourable environment for the colonization by *H. pylori*. Testing of *H. pylori *in the saliva and dental plaques in non cancer subjects wouldn't significantly add to the hypothesis as the site of colonization in betel chewers has to be the oral mucosa when compared to the non betel chewers in this study.

In this study we have detected *H. pylori *in the oral cavity in the absence of colonization in the stomach in oral cancer patients. We have no way of knowing if this represents residual colonization following an infection some time in the past or colonization primarily in the oral cavity. In either case it is possible that betel chewers, because of their greater risk of colonization by *H. pylori *may then be at risk of relapse or re-infection of gastric and duodenal infection in the future. Apart from the risk of oral cancer, betel chewing may be an additional risk in the Sri Lankan population for the development of peptic ulcer disease and gastric carcinoma.

## Competing interests

The authors declare that they have no competing interests.

## Authors' contributions

NF and NP conceived of the study, and participated in its design and coordination and helped to draft the manuscript. GJ participated in sample collection laboratory processing and analysis of data. (this study was submitted as partial fulfilment of a thesis submitted for a BSc Special Microbiology, University of Sri Jayewardenapura for GJ Kumar) IA provided samples and intellectual input in drafting the manuscript JH provided intellectual input and helped draft the manuscript. FM participated in laboratory processing and in drafting the manuscript. All authors read and approved the final version of the manuscript.

## Pre-publication history

The pre-publication history for this paper can be accessed here:


